# Visual Snow Syndrome as a Network Disorder: A Systematic Review

**DOI:** 10.3389/fneur.2021.724072

**Published:** 2021-10-04

**Authors:** Antonia Klein, Christoph J. Schankin

**Affiliations:** Department of Neurology, Inselspital, Bern University Hospital, University of Bern, Bern, Switzerland

**Keywords:** visual snow syndrome, systematic (literature) review, perceptual disorder, electrophysiology, imaging, network disorder, neuroophtalmology, visual disturbance

## Abstract

**Aim:** By reviewing the existing clinical studies about visual snow (VS) as a symptom or as part of visual snow syndrome (VSS), we aim at improving our understanding of VSS being a network disorder.

**Background:** Patients with VSS suffer from a continuous visual disturbance resembling the view of a badly tuned analog television (i.e., VS) and other visual, as well as non-visual symptoms. These symptoms can persist over years and often strongly impact the quality of life. The exact prevalence is still unknown, but up to 2.2% of the population could be affected. Presently, there is no established treatment, and the underlying pathophysiology is unknown. In recent years, there have been several approaches to identify the brain areas involved and their interplay to explain the complex presentation.

**Methods:** We collected the clinical and paraclinical evidence from the currently published original studies on VS and its syndrome by searching PubMed and Google Scholar for the term visual snow. We included original studies in English or German and excluded all reviews, case reports that did not add new information to the topic of this review, and articles that were not retrievable in PubMed or Google Scholar. We grouped the studies according to the methods that were used.

**Results:** Fifty-three studies were found for this review. In VSS, the clinical spectrum includes additional visual disturbances such as excessive floaters, palinopsia, nyctalopia, photophobia, and entoptic phenomena. There is also an association with other perceptual and affective disorders as well as cognitive symptoms. The studies that have been included in this review demonstrate structural, functional, and metabolic alterations in the primary and/or secondary visual areas of the brain. Beyond that, results indicate a disruption in the pre-cortical visual pathways and large-scale networks including the default mode network and the salience network.

**Discussion:** The combination of the clinical picture and widespread functional and structural alterations in visual and extra-visual areas indicates that the VSS is a network disorder. The involvement of pre-cortical visual structures and attentional networks might result in an impairment of “filtering” and prioritizing stimuli as top-down process with subsequent excessive activation of the visual cortices when exposed to irrelevant external and internal stimuli. Limitations of the existing literature are that not all authors used the ICHD-3 definition of the VSS. Some were referring to the symptom VS, and in many cases, the control groups were not matched for migraine or migraine aura.

## Introduction

The first description of the visual snow phenomenon (VS) was presented by Liu et al. in 1995 with four patients with migraine who had interictal visual disturbances (1). Initially, these symptoms were thought to be persistent migraine aura (2, 3). Through the collection of 22 patient reports, Schankin et al. (4) noticed that the symptoms are very characteristic and not as polymorphous as they can be in typical migraine aura (5, 6). This was confirmed in larger groups via an internet survey (235 persons) and an additional semi-structured telephone interview (142 persons with self-assessed VS) (4, 7). It became evident that this disorder also affects patients who have not been diagnosed with migraine (4, 8). Finally, the group concluded that the symptom VS is often associated with additional visual, non-visual, and non-perceptual symptoms forming the visual snow syndrome (VSS), which is distinct from migraine. Based on this, diagnostic criteria have been proposed (4) that have been implemented in the international classification of headache disorders, 3rd edition (ICHD-3) (4, 9). The aim of this review is to summarize what we have learned about the underlying pathophysiology of VSS from clinical presentation, secondary forms, neurobehavioral studies and imaging, as well as electrophysiological testing.

## Methods

We performed a systematic review on PubMed (accessed May 1, 2021) and Google Scholar (accessed May 15, 2021) using the search term “visual snow.”

We included case reports and studies describing epidemiological, neurophysiological, and imaging findings about the VSS or VS. We excluded articles not written in English or German, non-original work, such as reviews, case reports not adding information to the question of this review, and papers that could not be retrieved via Google Scholar or PubMed.

The records were screened by AK and also evaluated by CJS in respect of the inclusion and exclusion criteria.

Papers released prior to the publication of the diagnostic criteria for the VSS by the International Headache Society in 2018 (9) were not excluded when the criteria could be assessed retrospectively based on the clinical information presented.

Additionally, we performed a non-systematic search for the terms “palinopsia,” “floaters,” “blue field phenomenon,” “selflight of the eye,” “photopsia,” “Nyctalopia,” “Photophobia,” “Lamotrigine” AND “migraine aura,” “thalamostriatal loop,” “thalamocortical dysrhythmia,” and “salience network” to provide definitions of these expressions used in our review.

## Results

**Figure 1:**
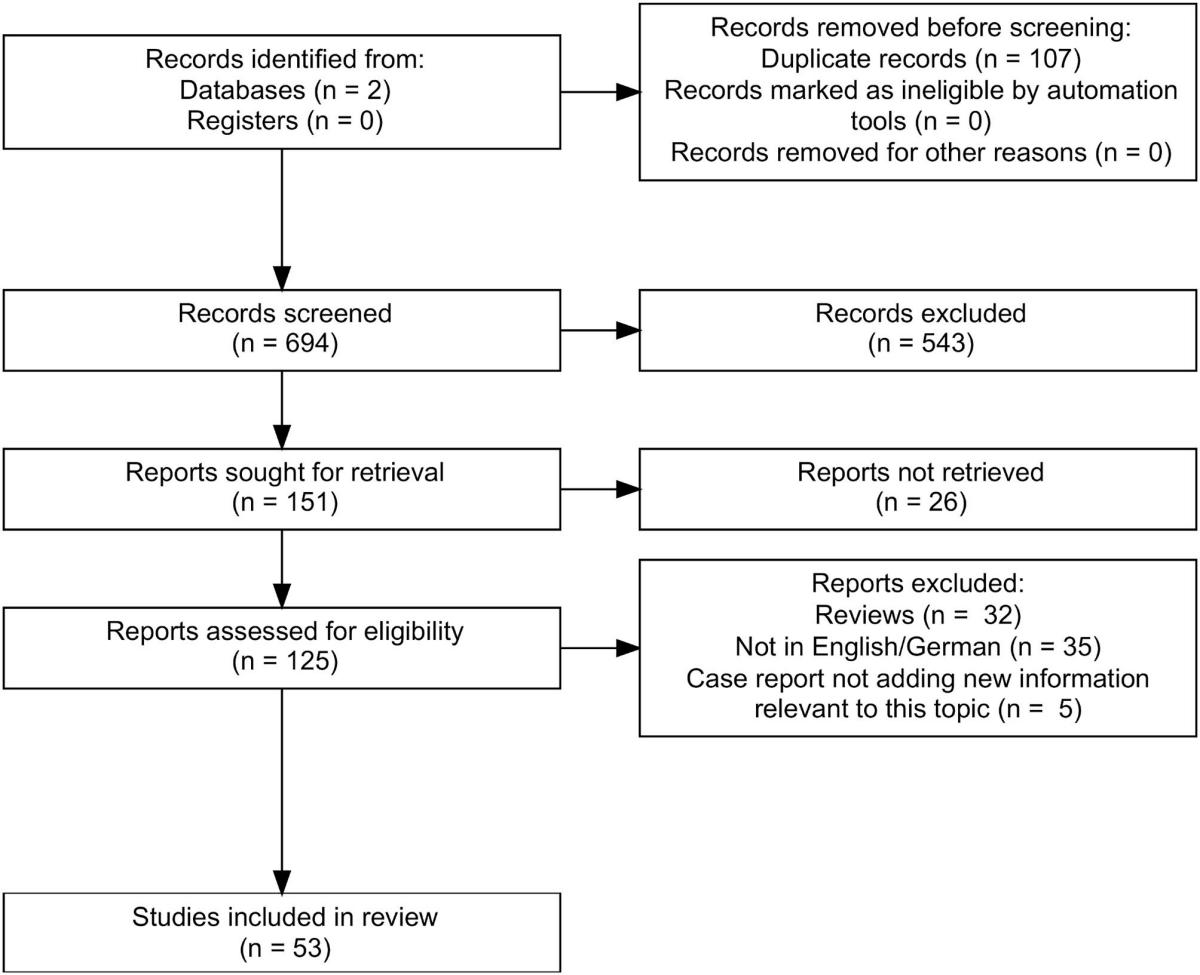
Flow chart reporting the search strategy according to PRISMA (10). We searched PubMed and Google Scholar and found 801 papers. After excluding doubles, there were 694 results left, of which 543 were not on the topic of this review. We finally retrieved 125 publications, of which 32 were reviews, 35 were not in English or German, and 5 case reports did not add new information to our topic. Thus, 53 articles were included in this systematic review.

In total, we found 801 papers. After excluding doubles (*n* = 107) and articles that were not on the topic (*n* = 543) of this review, there were 151 remaining of which 125 could be retrieved. Finally, we included 53 articles (Figure 1). We organized the articles according to the main topic and the used methodology.

### The Clinical Picture

Several studies have collected data on the prevalence of symptoms in groups of patients with VSS or VS, either in online surveys (4, 11, 12) or in interviews at headache centers or neuro-ophthalmologic departments (4, 13–17). Per definition, patients with VS experience a visual static, similar to the flickering of an old TV, with many, moving colored or black-and-white dots in the entire visual field. To fulfill the diagnostic criteria of the ICHD-3, there must be at least two additional characteristic symptoms:

1. Palinopsia is an abnormal, continued perception of an object even after it is no longer in the visual field (18, 19). This symptom is reported in 33% (14) to 86% (4) of patients with VSS. it can manifest as a steady afterimage or as “trailing,” i.e., positive afterimages persisting directly after a dynamic object (20).
2. Another group of symptoms are enhanced entoptic phenomena, which are thought to be perceptions of endogenic structures/phenomena of the eye (21):
  - Floaters can be caused by degenerative changes in the vitreous body including liquefaction (22, 23). According to the above-mentioned studies, between 61% (16) and 100% (24) of VSS patients are perceiving them in an excessive way, i.e., far more than normal vitreous floaters and far more than expected from ophthalmological findings.
  - The so-called “blue field phenomenon” is characterized by a perception of moving fragments against a bright background, such as the sky (25). It is the autovisualization of the own leukocytes in the retinal capillaries (26). It has been shown that about 42% (27) to 79% (4) of VSS patients experience this symptom, also in an excessive magnitude.
  - The “selflight of the eye” is light or colored clouds seen with closed eyes (26) and perceived by 16% (16) to 71% (11) of VSS patients. The cause of this phenomenon is unknown. Bowen et al. hypothesized that it might be linked to retinal circulation (26).
  - Photopsia, reported in 24% (15, 16) to 63% (4, 11) of VSS patients, are “flash-like” positive visual phenomena with sudden onset and brief duration (28). The origin in different diseases can be generated by almost all components of the visual path from ocular mechanical disturbances to affections of the secondary visual pathways (28).
3. Nyctalopia: Patients complain about poor vision in darker environments. In the literature, it has often been described in patients with retinal diseases (especially affecting the rod cells), but the inability to adapt to light conditions might involve almost all components of the visual pathway (29). About 28% (16) to 78% (11) of VSS patients are affected.
4. Photophobia, which is experienced by 44% (16) to 81% (11), is hypersensitivity, discomfort, or even pain caused by “normal” light (27, 30). Eren et al. (27) compared a group of 19 VSS patients to a group of 19 controls matched for age, sex, migraine, and aura using the Leiden Visual Sensitivity Scale (31). They demonstrated that VSS patients had increased visual sensitivity at a level comparable to patients with chronic migraine during their attacks (27).

### Prevalence and Course of Disease

Graber et al. conducted the first longitudinal study in VSS. Symptoms were persistent, in some cases up to 8 years (32). In several studies, there was a fraction of patients reporting VSS for as long as they could remember (4, 11, 14). The data so far suggest that the VSS does often become a chronic disease. There are also published cases of episodic VS as part of a migraine attacks, but different from aura (33).

The first study to assess the prevalence of VSS within the population was by Kondziella et al. who used an online survey with 1,015 participants. Although this study has its limitations (34), a larger proportion of the general population, 2.2% in this study, might fulfill the criteria of VSS (35).

### Associated Symptoms and Disorders

Between 52% (13) and 72% (11) of patients with VSS also suffer from migraine. In several clinical studies, patients reported migraine attacks in association with the appearance or the aggravation of their VSS (4, 11, 14, 16, 36). Schankin et al. (4) and Puledda et al. (11) explored the relationship between migraine status and the manifestation of VSS and found that people with migraine tended to have more symptoms as a marker of a stronger affection by this disease. Another highly prevalent comorbidity affecting between 15% (14) and 75% (11) of patients is tinnitus. Puledda et al. showed that for the VSS patients in their cohort who were also affected by tinnitus, the probability of having additional visual symptoms was 2 fold increased (11). Mehta et al. reported that 7.1% of their cohort with VS had a diagnosis of fibromyalgia (16). Additionally, postural orthostatic tachycardia syndrome (16), dizziness (16), balance problems (15), paresthesia (37), and tremor (15) have been described, but the latter without a further differentiation of the subtype.

Psychiatric comorbidities seem to be quite common in VSS, especially affective disorders such as anxiety, up to 50% (13), and depression, up to 58% (13). Patients also report cognitive complaints in the form of “brain fog” (16) and concentration problems (4). Another frequent symptom is derealization (16), which can be linked to the above-mentioned psychiatric disorders or appear independently (38).

### Secondary Forms of VS and Triggers

Ophthalmological or radiological routine findings are without pathological findings in most cases (4, 11, 16, 17, 39). There are some case reports of patients with positive visual phenomena similar to those in VS, which turned out to be caused by ocular pathology, such as birdshot retinopathia (16, 40), but also diseases leading to an extensive visual impairment like in a patient described by Mehta et al. who had advanced macular atrophy with cystic retinal degeneration and developed Charles-Bonnet syndrome with VS in addition (16). This might indicate that sensory deafferentation could play a role in the development of VS.

Cerebral diseases, especially affecting the occipital and/or temporal brain regions, such as a pinealis cyst (with amelioration of symptoms after removal) (41), a case of Creutzfeldt–Jakob disease (42), idiopathic intracranial hypertension, posterior cortical atrophy, multiple sclerosis (16), or glutamic acid decarboxylase antibody syndrome (43), have been described to cause secondary VS or even VSS. Catarci reported one patient who developed permanent left-sided VS in the context of an acute occlusion of the right posterior cerebral artery (44).

In some patients, systemic infections, seizures of probable temporal or occipital origin (16, 45), concussions (16, 45), hormonal changes, drugs such as steroids (16), antidepressants (46), or isotretinoin (47) were suspected triggers for VSS.

An important differential diagnosis is “hallucinogen persisting preception disorder” (HPPD): It is a chronic syndrome characterized by a spontaneous recurrence of perceptual/visual disturbances that are similar to those generated during an intoxication with a hallucinogenic drug (DSM V) (48). In the cohort of Puledda et al. (11), there were no significant differences concerning the phenotype of the VSS between a HPPD group (70 patients) and the VSS group (1,061 patients). The HPPD patients had similar comorbidities (migraine and tinnitus) (11). Van Dongen et al. did not find a difference in VSS manifestation (intensity) in a group of 24 HPPD patients and 37 VSS patients, but the VSS patients were significantly more affected by migraine (49). Drugs that were reported to cause VSS in HPPD were ecstasy, cannabis, psilocybin mushrooms, amphetamine, 4-FMP, 3-MMC, 2C-B, ketamine, and nitrous oxide (49). This is important since these recreational drugs can obviously “trigger” a VSS-like disorder in subjects who *per se* do not have increased risk due to the non-increased prevalence of migraine and migraine aura.

### Evidence for Therapeutic Effect of Medication

The current data indicate a possible effect of lamotrigine, which is an anti-seizure medication also shown to be preventive in migraine with visual aura (50). It inhibits voltage-gated sodium and calcium channels in the central nervous system, restricting the firing rate of cortical neurons and thereby lowering cortical excitability (50). Fekete et al. reported a case of VSS with complete remission under the therapy with lamotrigine (51), while other studies found only a small effect (in the sense of an amelioration) in a small number of patients (13, 52). There are single case reports of a decrease in symptom severity after the use of mydriatics (53) and a remission under amitriptyline (54). Antipsychotic drugs (55), glucocorticoids, beta-blocker, acetazolamide (17), other antidepressants (56) and antiseizure medications, benzodiazepines, migraine prophylaxis, and even ketamine were tried with a few cases of partial improvement, but mostly no benefit (16).

### Neurobehavioral Measures

Since the diagnosis of the VSS is at the moment solely based on subjective measures, there have been several attempts to find a specific neurobehavioral or electrophysiological signature. Solly et al. utilized oculomotor tasks to examine 64 VSS patients and 23 controls (one subgroup of VSS patients with migraine, one without migraine, and a healthy control group). They demonstrated significantly quicker prosaccades in VSS patients as well as more errors in incongruencies between precue and target (37). A follow-up study with 67 patients examined interfering or conflicting saccade tasks (of which one was internally cued) again showing quicker prosaccadic movements and more errors in choosing (also internally cued) antisaccadic (contralateral) marks. This suggests that the underlying problem might go beyond the management of attention given to external stimuli, but rather include a coordination problem on the level of the saccadic control system (with hyperactivity in the prosaccade system) (57).

Three groups examined visual perception thresholds in VSS patients demonstrating decreased spatial contrast sensitivity (17), reduced center-surround contrast suppression, elevated luminance increment thresholds on a textured background (58), and significantly more difficulties recognizing image orientations specifically at a flickering frequency of 15 Hz (59).

In another study, VS patients showed normal color and rapid flicker sensitivity but a delayed dilatation after the initial constriction of the pupil after the presentation of a chromatic stimulus (60). The latter could indicate a longer afferent stimulus persistence (60) or an autonomic dysregulation (17). A limitation of this study (60) was the small number of patients and controls included.

### Electrophysiological Evidence

As an approach to study the visual pathway, several groups recorded visual evoked potentials. Eren et al. compared a group of 18 VSS patients to matched healthy controls and migraine patients. They demonstrated an increased N145 latency, which could indicate a disturbance in the secondary visual areas. There were also reduced N75-P100 amplitudes representing the afferent visual pathways between the retina and the primary visual cortex (61). Two groups investigated habituation of the P100-response after repetitive stimulation in VS patients compared to healthy controls. Yildiz et al. included a subgroup of VSS patients with migraine and a subgroup of VSS patients without migraine (24), while Luna et al. examined only one VSS patient who did not have the diagnosis of a migraine (62). In both papers, decreased habituation indicated increased cortical excitability over the afferent visual pathways, especially the primary visual cortex (V1) and possibly a disturbance of negative feedback mechanisms (24, 62).

In an occipital TMS application, the phosphene threshold was lower in the VSS group compared to healthy controls (24). These findings, too, might indicate neuronal hyperexcitability in the visual pathway (24). In contrast, Eren et al. could not find a significant effect of TMS application over the visual cortex on letter recognition (63). Grey et al. applied TMS at 10 and 10 + 1 Hz over occipital brain areas during 20 sessions in nine patients. They found an improvement of VS intensities after the 10 + 1 Hz application, but no significant difference of the comparison to Sham or 10 Hz (64).

### Brain Imaging

Another attempt to find the causes underlying the VSS is looking for functional and structural correlates in neuronal imaging studies. Schankin et al. combined [^18^F]-2-fluoro-2-deoxy-D-glucose positron emission tomography (FDG-PET) in 20 VSS patients (matched with 20 healthy controls) with MRI in 17 patients and controls (65). Puledda et al. performed MRI (magnetic resonance imaging) with seed-based MR spectroscopy in 24 patients and an equal number of matched controls (66–68). Aldusary et al. compared a cohort of 19 VSS patients to 16 controls using MRI (69). In all three studies, patients and controls were matched for age and sex but not migraine (Table 1).

**Table 1:** Summary of the imaging findings.

**Modality**	**Study**	**Patients/ controls**	**Main findings**
FDG-PET	Schankin et al. (34)	20/20	• Hypermetabolism in the right lingual gyrus • Hypometabolism in the right superior temporal gyrus and the left inferior parietal lobule
MRI -Volumetry	Schankin et al. (34)	17/17	*Global analysis:* • GMV increased: junction of the right lingual and fusiform gyrus, right middle temporal gyrus, right parahippocampal gyrus, left superior temporal gyrus, right anterior cingulate cortex • GMV decreased: left superior temporal gyrus
	Puledda et al. (11)	24/24	*Whole brain morphology, parcellated cerebellar analyses, ROI-analyses:* • GMV increased: left V1(WB), left V1/V2 area (ROI), left V5 area (ROI), crus I/lobule VI of the left cerebellar hemisphere
	Aldusary et al. (69)	19/16	*Voxel-based morphometry:* • GMV increased: right lingual gyrus, visually: occipital bending in 7 patients
fMRI	Puledda et al. (11)	24/24	*MRI block-design (visual stimulation) with MRS:* • Reduced BOLD responses: left and right anterior insula, MRS lactate-Peak over right gyrus lingualis with anticorrelation to BOLD response
	Puledda et al. (68)	24/24	*fMRI seed-based connectivity analysis:* • Resting state (hyperconnectivity) - right pulvinar (PV)- right postcentral, supramarginal gyrus (SMG) - Pre-cuneus–right pre-central gyrus/frontal eye fields -V1–SMG and post-central gyrus • Resting state (hypoconnectivity): -right V5–posterior cingulate cortex. -cerebellar seed- PCC and medial precuneus -PV- bilateral caudate nuclei • Task (hyperconnectivity) - right PV-right lingual gyrus - right V1- right V5, postcentral,precentral gyri, SMG, premotor cortex, supplementary motor cortex(SMA), FEF -V5–right cuneus, Brodmann 17, 18 and 19, the FEF, SMG, premotor cortex, SMA, superior parietal lobule (SPL) and intraparietal sulcus, V1 -pMCC/PCC–bilateral medial pre-cuneus, PCC. -cerebellar seed–RSPL, lat pre-cuneus, post-central gyrus • Task (hypoconnectivity) -V5–posterior cingulate cortex, bilat medial pre-cuneus, TPJ und AG
	Aldusary et al. (69)	19/16	*Resting state fMRI (seed-based):* • *Hyperconnectivity:* -Left anterior inferior temporal gyrus–left posterior temporal fusiform gyrus -Right anterior inferior temporal gyrus–right anterior temporal fusiform gyrus -Left posterior superior temporal gyrus–right inferior occipito-temporal gyrus -Left angular gyrus–left lateral pre-frontal cortex -Right frontal eye field–right angular gyrus -Left inferior frontal gyrus–left middle frontal gyrus
[^123^I]-IMP SPECT	Shibata et al. (74)	3 patients/no controls	[^123^I]-IMP single-photon emission computed tomography 1. Right temporooccipital hypoperfusion 2. Mild bilateral frontal hypoperfusion 3. No pathological findings

In the FDG-PET analysis, focal hypermetabolism was demonstrated in the secondary visual area of the right gyrus lingualis presumably as a correlate of neuronal hyperactivity in this area (65). A matching increase in gray matter volume (in MRI volumetry) was shown in the adjacent right fusiform gyrus, and in the lingual gyrus itself in the cohort of Adusary et al. (69). Interestingly, the symptom duration positively correlated with gray matter volume (GMV) in both lingual gyri (69). This might reflect differences caused by neuronal plasticity (70). Additionally, the seed-based MR spectroscopy by Puledda et al. showed a lactate peak in this area correlating negatively with the BOLD response as a sign of anaerobic (possibly inefficient or abnormal) metabolism (67).

There are hints that other areas of the visual system might be implicated as well. Puledda et al. found increased gray matter volume (whole brain voxel-wise volumetry) in the left primary (seed-based) and secondary visual cortex (V2 and V5) (66). Beyond that, the group showed in a seed-based functional MRI (fMRI) analysis of the regions of interest in the right hemisphere that there was increased connectivity between the thalamus and the lingual gyrus at tasks while the connection between thalamus and basal ganglia resting state was decreased. V1 and V5 showed hyperconnectivity between each other and with widespread cortical regions (somatosensory and motor areas including the supramarginal gyrus and frontal eye field) during stimulation. V5 had a decreased connectivity to the posterior cingulate cortex at rest, which is part of the default mode network (68). Aldusary et al. found seed-based fMRI resting state hyperconnectivity between extrastriate visual and other temporal brain regions (69).

Other non-visual brain areas seem to be affected as well. The response to a stimulation mimicking VS resulted in a reduced BOLD response compared to baseline over the bilateral anterior insulae (67, 71). Aldusary showed resting state hyperconnectivity between pre-frontal and parietal brain regions (69). Schankin et al. demonstrated FDG hypometabolism in the right superior temporal gyrus and the left inferior parietal lobule without associated structural alterations (65). Volumetrically, increased gray matter volume in the right middle temporal gyrus, parahippocampal gyrus, the left superior temporal gyrus and right anterior cingulate cortex (65), and cerebellum (seed-based) crus I/lobule V of the left hemisphere (66) was found.

There is one case report of a patient with VSS and another one about a patient with a prolonged migraine aura with VS demonstrating increased diffusivity in the occipital lobe, as well as the temporal lobes including the dorsal visual stream, the ventral visual stream, and the integrative visual stream (72, 73). Shibata et al. performed [^123^I]-IMP single-photon emission computed tomography in one VSS patient showing right occipital and temporal hypoperfusion with and minimal bifrontal hypoperfusion in a second case (74).

## Discussion

The clinical picture of VSS consists of a characteristic constellation of visual symptoms that might be attributed to different components of the visual pathway.

In patients with migraine, the involvement of the right lingual gyrus has been shown in photophobia (75). In VSS, we find imaging, metabolic, and possibly electrophysiological evidence of increased metabolism, excitability, and connectivity in and with this area. The fMRI findings by Puledda et al. (68) indicate an implication of the visual motion area V5 with increased connectivity within and beyond the visual cortices with multiple brain areas. These give further insights into the pathophysiological mechanisms underlying the VS phenomenon and could be connected to the dynamic nature of this visual misperception (68).

The perception of entoptic phenomena indicates a “filtering” problem since these partly “physiological” but irrelevant sensations are enhanced in VSS (22).

On another level, nyctalopia and the increased luminance threshold (58) and decreased contrast sensitivity (17) might be connected by a lack of inhibitory feedback mechanisms in the visual system, normally allowing to extract relevant information and suppress visual noise.

Palinopsia might also be a phenomenon of disinhibition, leading to the repetitive perception of a dysfunctional visual memory (76). In this regard, previous case reports show that palinopsia can be caused by occipital, parietal, or temporal lesions mostly of the right hemisphere (76).

The electrophysiological evidence suggests hyperexcitability of the primary visual cortex (24, 61) possibly explaining the perception of photopsia.

Puledda et al. found in their fMRI analysis a disturbance in thalamostriatal connectivity while thalamocortical connections were increased (68). The thalamostriatal loop was shown to be relevant in visual learning (77), and it was demonstrated in an animal model that a disruption of this system leads to a decrease in visual precision (68, 77). The thalamus is linked to widespread areas of the cortex including the primary and secondary visual areas of the brain (78). This is consistent with the hypothesis that VSS could be a form of thalamocortical dysrhythmia (15).

On the other hand, the decreased BOLD response over the anterior insulae (67) as well as the increased gray matter volume over the right anterior cingulate cortex (65) could indicate a disturbance in the so-called salience network. This network plays a central role in the steering of attention, coordination between large-scale networks in task-related functions (79), and the selection of relevant stimuli (80). Furthermore, the main hubs of the salience network and the thalamus are closely interconnected (81).

There are some limitations of studies on VSS that have to be considered also in this review. Many of the earlier studies were done in smaller groups. For many imaging and most electrophysiological findings, patients have not been matched for migraine or migraine aura. This is relevant since a high comorbidity would confound the findings by also investigating migraine instead of VSS alone. In some of the case reports and neurobehavioral tests, the ICHD-3 criteria for VSS have not been applied. Similarly, patients might have been affected by the symptom VS, instead of VSS. Furthermore, intake of medication or previous drug use has not been reported in all studies. Future studies have to apply the ICHD 3 criteria for VSS and should include a reasonably sized number of patients that are matched for migraine and migraine aura. To study pharmacological and non-pharmacological treatment approaches, randomized and controlled trials are needed.

## Conclusion

Already from a clinical perspective, the symptoms of VSS cannot be attributed to a single brain area or a functional unit. Rather, there is evidence of a network disorder that might manifest as a disturbance in coordination or interaction between different parts of the visual system causing a loss of inhibitory modulation and thereby hyperactivity in the primary and secondary visual cortex.

VSS seems to be a spectrum disorder with different degrees of severity, e.g., defined by the number of additional symptoms (11). There is an association with tinnitus thought to be caused by cortical hyperexcitability and production of a phantom sensation (82). Several patients may suffer from comorbid fibromyalgia, the classical centralized pain disorder with hypersensitivity to external stimuli (83). There are potentially common underlying mechanisms (doi: 10.1111/head.14213).

Migraine is the most prevalent comorbidity in patients affected by VSS. Migraine can be seen as a sensory gating disorder with a persisting hypersensitivity to internal and external stimuli even in the interictal state (84, 85). It is associated with more severe manifestations of VSS. It can be hypothesized that migraine could cause a pre-disposition to develop a persistent sensory network disorder like VSS or, based on case reports, migraine attacks could even be triggers.

Other triggers include medications, seizure, trauma, and recreational drug use, when HPPD is counted as a form of secondary VSS. Secondary VSS caused by structural lesions seem to involve different posterior areas of the brain. One potential mechanism of secondary and triggered forms might be an imbalance of this network.

Understanding the communication within this network and how its modulation might lead to VSS is crucial if treatment strategies should be developed for this currently almost untreatable condition.

## Data Availability Statement

The original contributions presented in the study are included in the article, further inquiries can be directed to the corresponding author.

## Author Contributions

AK: conceived and designed the research, collected the data, organized the papers, and wrote the paper. CJS: conceived and designed the research, organized the papers, and wrote the paper and checked the manuscript. All authors contributed to the article and approved the submitted version.

## Funding

This research was funded by the Visual snow Initiative: funding of the publication fee. CJS was supported by Baasch Medicus Foundation.

## Conflict of Interest

CJS received scientific support, travel support and/or honoraria from Novartis, Eli Lilly, TEVA Pharmaceuticals, Lundbeck, Allergan, Almirall, Amgen, MindMed, Grünenthal. He received research grants from German Migraine and Headache Society, Eye on Vision Foundation, and Baasch Medicus Foundation. CJS is part-time employee at Zynnon. The remaining author declares that the research was conducted in the absence of any commercial or financial relationships that could be construed as a potential conflict of interest.

## Publisher's Note

All claims expressed in this article are solely those of the authors and do not necessarily represent those of their affiliated organizations, or those of the publisher, the editors and the reviewers. Any product that may be evaluated in this article, or claim that may be made by its manufacturer, is not guaranteed or endorsed by the publisher.
